# Clinical outcomes in early rehabilitation using the Össur Power Knee: An exploratory pilot study

**DOI:** 10.33137/cpoj.v9i2.47124

**Published:** 2026-07-08

**Authors:** S Schaeffer, K Bufe, S Kluge, M Blaschke, N Linnert, K Lechler, A.P Schulz

**Affiliations:** 1. Department of Clinical Research, BG Klinikum Hamburg, Hamburg, Germany.; 2. Department of Rehabilitation Medicine, BG Klinikum Hamburg, Hamburg, Germany.; 3. Department of Physiotherapy, BG Klinikum Hamburg, Hamburg, Germany.; 4. Óssur ehf, R&D, Medical Office, Grjótháls 5–7, 110 Reykjavík, Iceland.

**Keywords:** Powered Prosthetic Knee, Early Rehabilitation, Transfemoral Amputation, Functional Mobility, Outcome Measures, Amputation, Functional Ability

## Abstract

**BACKGROUND:**

Early rehabilitation after transfemoral amputation aims to restore mobility, reduce compensatory strain, and improve quality of life. Powered prosthetic knees, such as the Össur Power Knee, may facilitate more physiological gait patterns in comparison to conventional prosthetic knees; however, evidence regarding their use during the initial rehabilitation phase remains limited.

**OBJECTIVE:**

To explore the safety and usability of the Össur Power Knee during rehabilitation following transfemoral amputation.

**METHODOLOGY:**

This prospective single-center exploratory study was conducted in a rehabilitation center of a public trauma hospital in Germany from October 1st 2023 until July 1st 2024 and included three adults with unilateral transfemoral amputation, who received the Össur Power Knee III (2021) as their initial prosthesis and completed standard interdisciplinary rehabilitation. Assessments were performed at baseline, 14 days, 1, 3 and 5 months. Outcomes were assessed in five domains: pain assessment, health-related quality of life, objective mobility and functional capacity, balance confidence and functional independence as well as prosthesis usability. Safety and adverse events were monitored throughout follow-up.

**RESULTS:**

Over five months, all three participants demonstrated improvements in EuroQol 5-Dimension 5-Level, Locomotor Capabilities Index, and Amputee Mobility Predictor scores, while two participants experienced reduced pain. Prosthesis usability scores varied across participants. Functional performance measures remained stable or improved across all cases. Two minor falls occurred that were unrelated to the device and did not result in injury.

**CONCLUSION:**

Early rehabilitation using the Össur Power Knee was feasible. During rehabilitation, patients reported reduced pain and improvements in mobility and patient-reported outcomes. However, these outcomes cannot be solely attributed to the Össur Power Knee, as interpretation is limited by the small sample size, single-arm design, and short follow-up period.

## INTRODUCTION

Early rehabilitation is critical for optimizing functional outcomes following transfemoral amputation. While conventional mechanical and passive microprocessor-controlled knee (MPK) prostheses have demonstrated benefits in mobility and safety,^1^–^3^ limited evidence exists regarding the impact of early integration of powered prosthetic knees.

Powered prosthetic knees, such as the Össur Power Knee, represent a recent advancement in prosthetic technology.

Powered prosthetic knees are designed to promote a more natural gait pattern, reduce abnormal biomechanical loading on the musculoskeletal system, and lower the metabolic cost associated with walking. By incorporating motorized actuators, powered knees aim to restore some of the lost muscular function in individuals with transfemoral amputation.^4^

Clinical literature on powered lower-limb prostheses is still emerging with most literature only comparing or evaluating MPKs and non-microprocessor knees (NMPKs).^2,5^ Early studies suggest powered prosthetics can outperform passive devices in specific tasks. For example, in the study performed by Pasquina et al.,^6^ where four wounded warriors received a power knee prosthesis. The researchers stated that “*All patients achieved significant mobility milestones more rapidly than historical norms of similarly injured patients using other prosthetic knees*.” Reported advantages of powered knee prosthetics include improved performance during inclined walking, restoration of natural leg movements, enhanced hip and knee joint symmetry in sit-to-stand transitions and reduced compensatory effort from the sound limb during stair ascent.^7^–^9^

Additionally, there is evidence that experienced prosthetic users can adapt relatively quickly, sometimes within 20 minutes, to operating powered prosthetic systems.^6^ These studies mainly focused on prosthesis users who already had a high level of mobility and experience. Many of those studies examined advanced or even experimental setups, including powered systems combining both knee and ankle functions.^10,11^ In contrast, there is still limited insight into how powered prosthetic knees perform when introduced early in the rehabilitation process, especially in individuals who are completely new to prosthetic use.

Beyond their objective functional benefits, there is currently little to no evidence from patient-reported outcome measures following fitting with powered prosthetic knees. The impact of powered prosthetic knees on patients’ perceived mobility and balance, as well as on other outcomes such as pain, quality of life, and usability, is relevant for the overall evaluation of these devices.

This study aims to explore clinical outcomes associated with a powered knee as the initial prosthesis during rehabilitation, serving as a foundation for future controlled trials. This study takes a step toward filling that gap by examining whether the active mechanical assistance provided by a powered knee might help initiate mobility and contribute to early improvements in function and quality of life as well as reductions in pain during early rehabilitation. Improvements across these outcomes are expected throughout the five-month follow-up period.

## METHODOLOGY

This prospective, non-interventional pilot study investigated early rehabilitation outcomes in individuals receiving a powered knee prosthesis (Össur ehf, Power Knee™) as their initial prosthetic device following unilateral transfemoral amputation. This study was conducted without external funding, and the primary aim was to evaluate subjective and objective clinical outcomes following the introduction of powered prosthetic technology during the early stages of rehabilitation.

The provision of the powered knee prosthesis was not part of the study intervention but constituted an eligibility criterion for study participation. Patients received the powered knee as part of their routine clinical care, irrespective of study enrollment, and were able to receive the device without participating in the study. The study therefore prospectively observed outcomes in patients who had already been prescribed a powered knee and did not influence clinical decision-making or treatment allocation.

The study employed a single-arm, single-center case series with structured follow-up assessments over five months at five predefined timepoints. The baseline is defined as the day of fitting with the power knee joint. The patients received standard rehabilitation in accordance with the clinic’s protocol. This included early prosthetic fitting, progressive gait training, task-specific functional exercises, and adaptation to the active assistance provided by the powered knee during level walking, stair negotiation, ramp ambulation, and sit-to-stand activities. Rehabilitation also included patient-oriented pharmacological pain management according to the institutional protocol. Rehabilitation was delivered by an interdisciplinary team, integrating physiotherapy, occupational therapy, prosthetic management, and patient education to optimize prosthesis use, mobility, and community participation. Standard clinical assessment procedures involved regular evaluation of residual limb health, prosthetic fit, gait biomechanics, balance, walking endurance, and functional independence, with therapeutic adjustments based on patient performance.

The responsible prosthetists and physiotherapists were trained and experienced in the fitting and rehabilitation procedures associated with the provided powered knee prosthesis before study initiation, with this training conducted independently of the study. During the study no additional formal or informal training or clinical support was provided by the manufacturer. Contact to the manufacturer only consisted of the ordering of the powered knee prosthesis.

### Investigated Prosthesis

The Össur Power Knee III (2021) is a CE-marked, MPK equipped with motorized actuation to support a more physiological gait pattern compared to conventional knee prosthetics. It is primarily designed for individuals classified as K3 or higher, or for those with the demonstrated potential to reach this functional level, as defined by the Health Care Financing Administration (HCFA) Common Procedure Coding System (HCPCS, 2001).

### Inclusion and Exclusion Criteria

Patients were recruited from respective departments for the rehabilitation of amputees in our hospital. Screening was carried out via the hospital’s digital information system by trained study personnel. Recruitment took place from October 1st, 2023, until January 31st, 2024. Patients that fulfilled the inclusion criteria and did not exhibit any of the exclusion criteria were invited for participation by study personnel. Inclusion and exclusion criteria for this study are stated in **Table 1**.

**Table 1:** Inclusion and exclusion criteria for study participation.

**Inclusion**
Provided with the Össur Power Knee as their primary prosthesis
Age ≥ 18 years
Body weight between 50–116 kg (manufacturer specifications and physician determination)
No prior prosthetic experience (first-time prosthesis users)
Unilateral transfemoral amputation
Adequate cognitive ability to understand study procedures and complete questionnaires reliably
Positive rehabilitation prognosis with an expected AMPPRO score >28 by the 5-month follow-up (T5), as determined at baseline
Minimum vertical clearance of 37 mm between the mechanical knee center and the proximal pyramid dome
**Exclusion**
Known contraindications to Power Knee use (as per manufacturer’s IFU)
Pregnancy
Serious comorbidities impairing safe rehabilitation (e.g., unstable cardiovascular conditions, severe dementia)
Incompatible anatomical conditions or insufficient residual limb control (to be determined by study physician)
Exposure to extreme environmental conditions incompatible with device operation

Eligible patients received full explanation of study aims, procedures and potential risks from the study physician. Four patients were invited to participate. The responsible physicians and physiotherapists were responsible for the assessment of eligibility concerning the patient’s mobility and rehabilitation prognosis. Patients were only enrolled after obtaining their written consent.

### Timepoints

Patients were assessed at five key follow-up intervals:

- **T1:** Day 0 (Baseline): Initial assessment on the same day as the prosthetic fitting
- **T2:** Day 14
- **T3:** 1 Month
- **T4:** 3 Months
- **T5:** 5 Months: Final study assessment to evaluate outcomes and usability.

### Assessment Categories

All assessments (**Table 2**) were administered within routine clinical practice in a public trauma hospital during the patients’ rehabilitation to ensure ecological validity and minimize patient burden. The outcome measures consist of pain assessment via numeric rating scale, European Quality of Life 5 Dimensions 5 Levels (EQ5D-5L), Amputee Mobility Predictor (AMPPRO), 10 Meter Walk Test (10MWT), Timed Up and Go Test (TUG), 2 Minute Walk Test (2MWT), Timed Stair Test (TST), Activities-Specific Balance Confidence Scale (ABC-D), Locomotor Capabilities Index (LCI-5), System Usability Scale (SUS) and the Questionnaire for Persons with Transfemoral Amputation (Q-TFA).

**Table 2:** Description of Instruments.

Test/Questionnaire	Reference	Administered at	Description
Numeric Rating Scale (NRS) for pain assessment	Farrar et al., 2001^12^	T1 – T5	Subjective pain intensity was recorded with and without the prosthesis at each timepoint via validated 11-point pain intensity numerical rating scale (PI-NRS).
European Quality of Life 5 Dimensions 5 Levels (EQ5D-5L)	Herdman et al., 2011^13^	T1 – T5	A validated instrument assessing five dimensions of health-related quality of life in form of a questionnaire, providing a single index value of a person’s perceived quality of life.
Amputee Mobility Predictor (AMPPRO)	Gailey et al., 2002^14^	T1 – T5	A validated, objective instrument for the assessment of functional ambulation in lower-limb amputee subjects.
10 Meter Walk Test (10MWT)	Batten et al., 2019^15^	T3 – T5	A reliable, objective instrument to measure gait speed, even for prosthetic leg users, with higher gait speeds indicating better mobility.
Timed Up and Go Test (TUG)	Schoppen et al., 1999^16^	T3 – T5	A validated test measures the time a person takes to stand up from a chair, walk 3 meters back and forth and take a seat again, giving insights into their basic mobility and balance. Also applicable to patients with an amputation of the lower extremity.
2 Minute Walk Test (2MWT)	Brooks et al., 2001^17^	T3 – T5	The 2-minute walk test is a validated test measuring the distance a person walks within 2 minutes. It is also suitable for patients in rehabilitation after lower limb amputation.
Timed Stair Test (TST)	Nightingale et al., 2014^18^	T4 & T5	A reproducible mobility test that measures beyond basic mobility tasks. Stairs require greater range of motion and strength, they are part of advanced activities of daily living. This test measures the person’s ability and time needed to ascend and descend 2 flights of stairs.
Activities-Specific Balance Confidence Scale (ABC-D)	Schott, 2011^19^	T3 – T5	A reliable and valid instrument that captures self-reported confidence during daily activities.
Locomotor Capabilities Index (LCI-5)	Franchignoni et al., 2004^20^	T3 – T5	A questionnaire suitable for determining subjective global locomotor ability of people with lower-limb amputation during prosthetic training.
System Usability Scale (SUS)	Brooke, 1996^21^	T3 – T5	A reliable, 10-item, 5-point Likert scale questionnaire developed to measure user-perceived usability including hardware such as motorized prosthetics.
Questionnaire for Persons with Transfemoral Amputation (Q-TFA)	Hagberg & Branemark, 2004^22^	T3 – T5	A validated and reliable self-reported measure for nonelderly transfemoral amputees using a prosthetic, which reflects use, mobility, problems and global health.

Mobility tests were only conducted if patients had sustained sufficient mobility to complete these. The questionnaires ABC-D, LCI-5, SUS and Q-TFA were introduced at T3. Outcome measures included a combination of validated objective and subjective tools.

The outcome measures can be divided into five assessment categories: 1) pain assessment, 2) health-related quality of life, 3) objective mobility and functional capacity, 4) balance confidence and functional independence as well as 5) prosthesis usability. The respective tests and questionnaires are listed in the **Table 2**.

### Data Collection and Management

All data were collected in accordance with Good Clinical Practice (GCP) and institutional data protection standards. Data acquisition was embedded into routine clinical rehabilitation procedures and was carried out by trained clinical personnel under the supervision of the study team. Functional tests were conducted only when patients demonstrated sufficient capacity to perform them.

All data were initially collected using paper-based Case Report Forms (CRFs) during scheduled study visits. These forms included both objective performance measures (e.g., test scores, distances, times) and patient-reported outcomes (e.g., EQ5D-5L, SUS, ABC-D), as well as sociodemographic data, relevant clinical observations, prosthetic adjustments, and adverse events (AEs), including serious adverse events (SAEs). These were defined as follows:

#### AEs:

- Skin irritation, pressure sores, or blisters at the socket interface,
- Residual limb pain or swelling,
- Unusual muscle soreness or fatigue during rehabilitation,
- Minor falls or stumbles without injury related or unrelated to the device,
- Discomfort or difficulty adapting to the prosthesis,
- Prosthesis related issues that don’t cause serious harm (e.g., instability, improper fit).

#### SAEs:

- Falls, related or unrelated to the device, leading to fractures, head injury, or hospitalization,
- Severe skin breakdown or infection requiring medical treatment or surgery,
- Device malfunction causing significant injury,
- Any other events related or unrelated to the device resulting in hospitalization, long-term disability, or lifethreatening complications.

After data collection, data were transferred to a secure digital database. Any inconsistencies or missing values were reviewed, and only de-identified data were used for analysis to ensure participant confidentiality. The study-specific CRF is provided in the **Appendix**.

### Statistical Analysis

Given the exploratory nature and small sample size of this pilot study, descriptive statistical methods were employed to depict changes in patient outcomes over the five-month rehabilitation period. Protocol deviations, as defined in the study protocol, included incomplete datasets due to fatigue or medical constraints, interindividual variability in completion of mobility tests and inconsistent completion of assessments.

For the purpose of the investigation, the recorded parameters were anonymously transferred from the questionnaires to an Excel spreadsheet. Individuals not involved in the study won´t be able to connect the data to patients. The descriptive data processing and presentation is carried out with Microsoft Office Professional Plus 19 Excel.

To evaluate the longitudinal progression of patient outcomes over the rehabilitation period, the data is individually reported per patient and timepoint (T1 to T5) with line graphs. Given the exploratory nature of this case series and the very small sample size (n = 3), formal statistical significance testing was not performed.

**Ethics:** This study was approved by the Ethics Committee of the Hamburg Medical Association (Ärztekammer Hamburg), Weidestraße 122b, 22083 Hamburg, on April 27, 2023 (Reference No. 2023-101030-BO-ff). Written informed consent was obtained from all three participants.

## RESULTS

A total of three individuals were enrolled in this single-center pilot study. All participants had undergone unilateral transfemoral amputation and received the Össur Power Knee as their first prosthesis during early rehabilitation. Each participant met all inclusion criteria and completed the five-month follow-up without dropout. Although visit completion reached 100%, the datasets were not fully complete since some individual measurements were missing. This is depicted in the graphs below (**Figure 1 to 7**). The study cohort consisted of two male and one female participant, aged between 47 and 68 years. Etiologies of amputation included both trauma and chronic infection, reflecting a clinically relevant and heterogeneous patient population commonly encountered in rehabilitation settings (**Table 3**).

**Figure 1:**
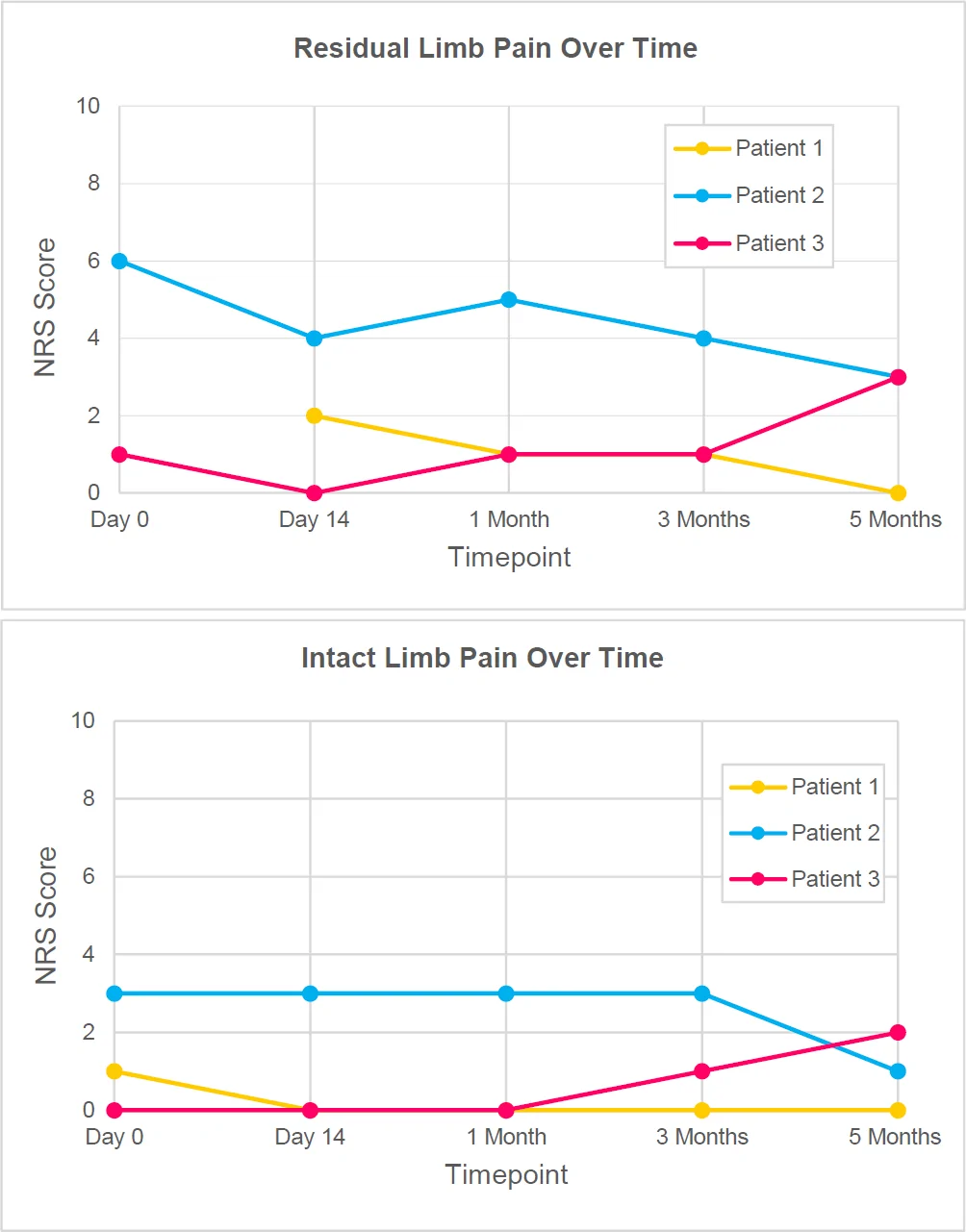
Longitudinal pain in three individuals following early fitting with a powered prosthetic knee.

**Figure 2:**
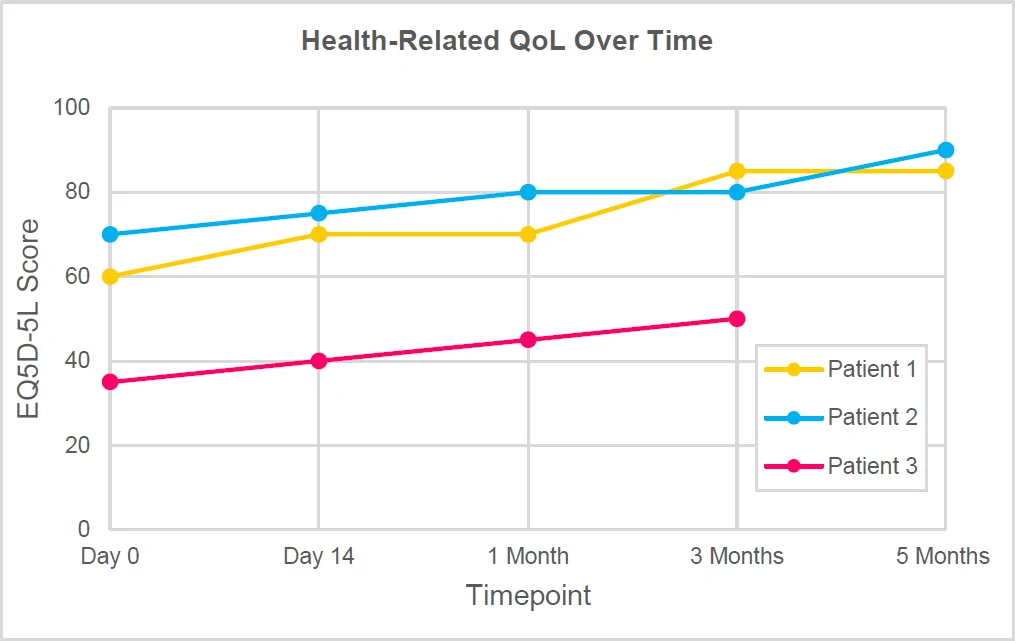
Longitudinal health-related quality of life in three individuals following early fitting with a powered prosthetic knee.

**Figure 3:**
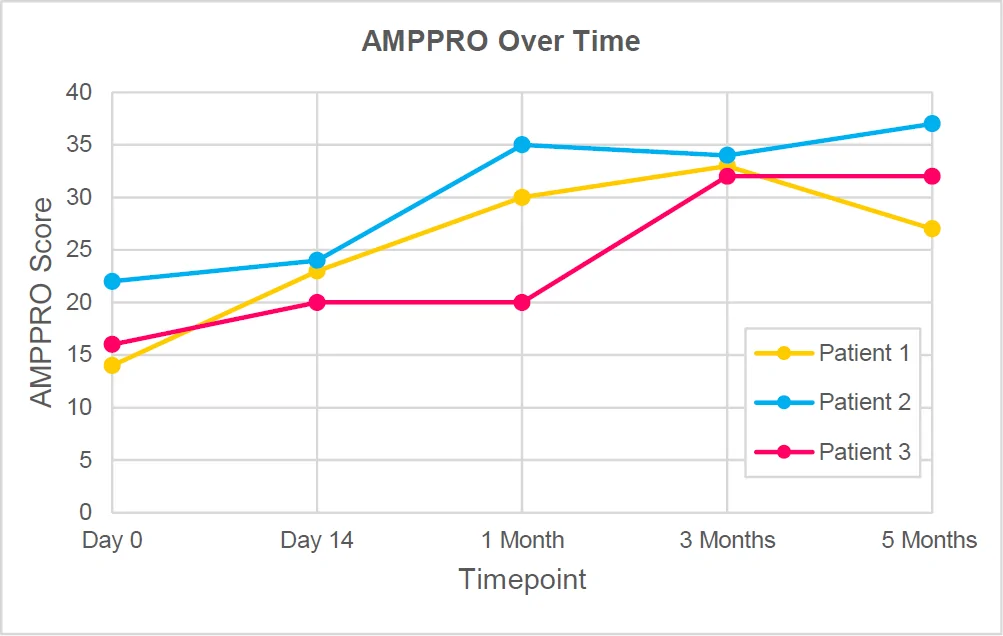
Longitudinal AMPPRO score in three individuals following early fitting with a powered prosthetic knee.

**Figure 4:**
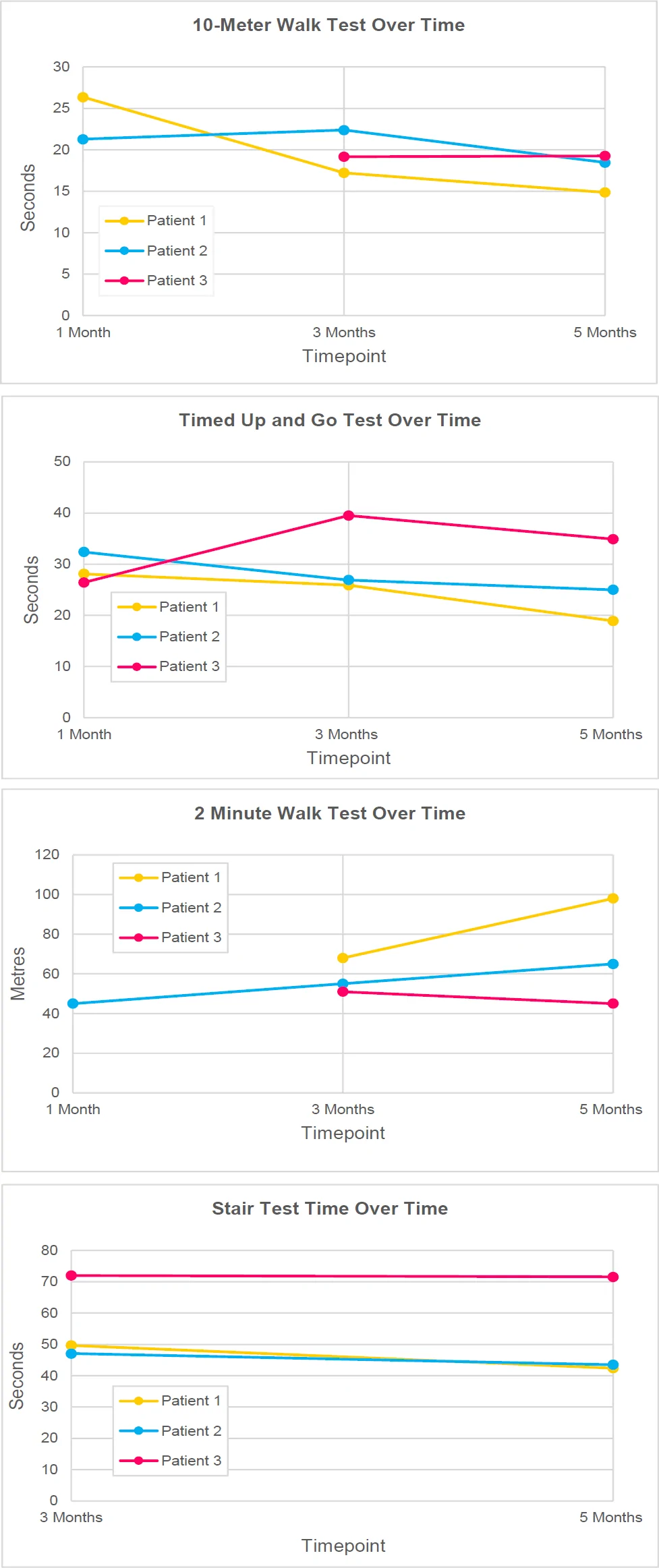
Longitudinal results of mobility tests in three individuals following early fitting with a powered prosthetic knee.

**Figure 5:**
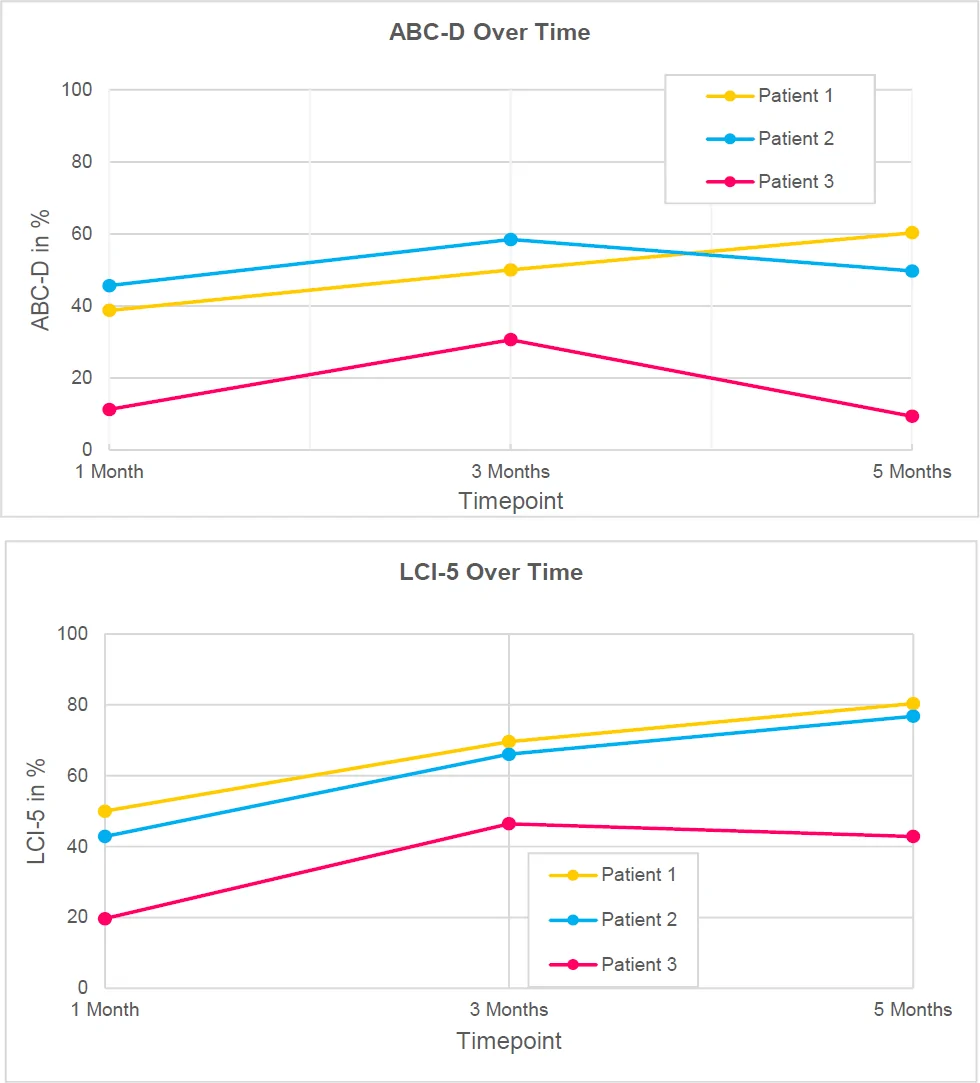
Longitudinal results of subjective mobility tests in three individuals following early fitting with a powered prosthetic knee.

**Figure 6:**
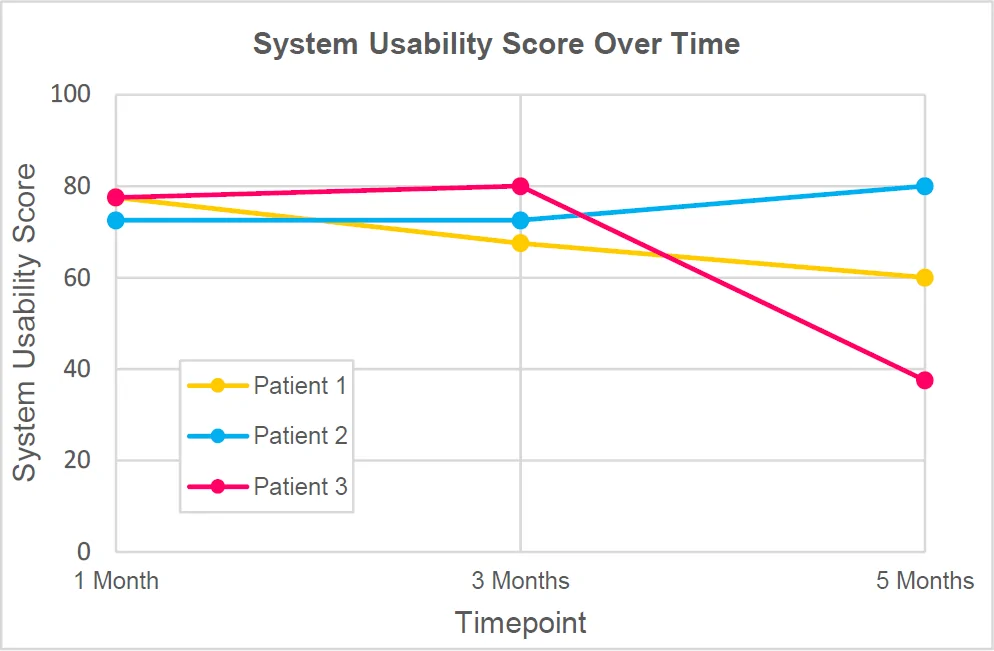
Longitudinal system usability score in three individuals following early fitting with a powered prosthetic knee.

**Figure 7:**
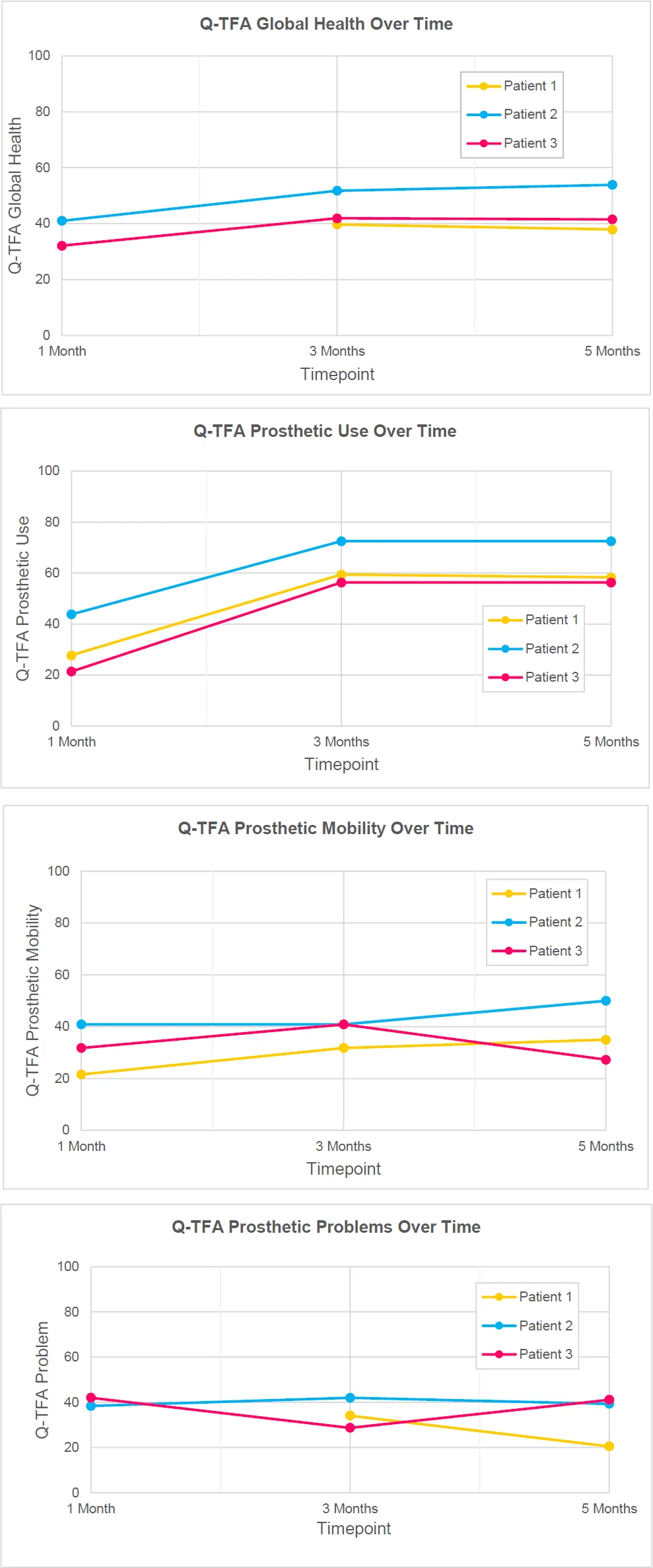
Longitudinal Q-TFA sub-scores (questionnaire for persons with a transfemoral amputation) in three individuals following early fitting with a powered prosthetic knee.

**Table 3:** Demographic Data of Included Patients.

Patient ID	Age	Gender	Amputation cause	Time from amputation to inclusion	Power Knee use in hrs/day at T5	Occupation
001	60	Male	Infection	35 days	7.2	Metalworker
002	47	Female	Trauma	48 days	7.2	Nurse/Geriatrics
003	68	Male	Infection	20 days	7.3	Forklift operator

In the duration of this case series no serious adverse events occurred. Two adverse events were reported, both minor falls without injury unrelated to the device.

### Pain Assessment

Pain was assessed via a numeric rating scale (NRS) from 1 to 10 for both the intact and amputated limb. The NRS pain scores for the limb with prosthesis showed a reduction in pain over time in two patients, indicating effective pain management throughout the rehabilitation phase. Patient 1 reported a decrease in pain from level 2 (T2) to 0 (T5) and patient 2 from 6 to 3. However, patient 3 reported a slight increase in pain from 1 to 3, throughout the observation period (**Figure 1**). Pain in the intact limb also depicted a trend toward improvement in two out of the three observed patients. The pain scores for the intact limb changed as follows: patient 1 reported a decrease in pain from 1 to 0, patient 2 from 3 to 1 and patient 3 reported a slight increase from 0 to 2 (**Figure 1**).

### Health-related Quality of Life

Subjective health-related quality of life (QoL) was assessed via the EQ5D-5L, which ranges from 0 to 100, 100 indicating the best possible QoL. An overall improvement was found in the EQ5D-5L health-related quality of life scores between baseline and five-month follow-up. The score demonstrated a consistent increase across all three patients over the observation period. Patient 1 reported a health-related QoL score increasing from 60 at T1 to 85 at T5, while patient 2 showed an increase from 70 at T1 to 90 at T5. Patient 3 exhibited an improvement from 35 at T1 to 50 at T4, the T5 value was not available as this datapoint was not documented for patient 3 at T5 (**Figure 2**).

### Objective Mobility and Functional Capacity

The AMPPRO score is an objective assessment tool used to evaluate the functional mobility and walking ability of lower-limb amputees while they are wearing their prosthesis. It ranges from 0 to 47, with 47 depicting the best possible functional mobility. All three patients reported an increase in mobility according to the AMPPRO throughout the entire observation period (T1 – T5). The score reported by patients 1 and 3 doubled over the observation period (patient 1: T1 = 14; T5 = 27; patient 3: T1 = 16; T5 = 32). Patient 2 reported an increase of over 50% from 22 at T1 to 37 at T5 (**Figure 3**).

The 10-Meter Walk Test was administered starting at T3 (or T4), as the therapists and/or physicians conducting the follow-up visits judged patients unable or unsafe to complete the test at earlier time points. The 10MWT measures the time needed for a patient to walk 10 Meters, subsequently a shorter time needed corresponds with better walking abilities. A major improvement was documented in patient 1, where the necessary time decreased from 26.35 sec to 14.86 sec from T3 to T5. Patient 2 depicts fluctuating results yet an overall decrease (T3: 21.3 sec, T4: 22.4 sec, T5: 18.5 sec) in time needed to complete the 10MWT. Lastly patient 3 was only able to complete the test at T4 and T5 with similar results (T4: 19.2 sec, T5: 19.3 sec) (**Figure 4**).

The Timed Up and Go (TUG) test was also only performed if patients presented capable without walking aids. It measures the time necessary for patients to stand up from a chair, walk 3 meters back and forth and sit back down. Subsequently less time needed indicates better mobility. While patient 1 and 2 depict positive advancements of nearly 10 seconds from T3 to T5, patient 3 shows fluctuating results with an overall increase in time necessary to complete the test. Patient 1 is able to complete the TUG test in under 20 seconds by the end of the observation period. Patient 2 completes the TUG in 25 seconds and patient 3 in 35 seconds (**Figure 4**).

The 2 Minute Walk Test examines the distance patients can walk in two minutes and is measured in meters. A longer distance travelled corresponds to better mobility. Patient 2 improved constantly from T3 (45m) and T4 (55m) to T5 (65m). Patients 1 and 3 only completed the test at T4 and T5, while patient 1 improved from 68 to 98 meters, yet patient 3 completes a longer distance at T4 (51) than T5 (45) (**Figure 4**).

Lastly, the Timed Stair Test measures the time to ascend and descend 2 flights of stairs. All 3 patients only underwent this test at T4 and T5 as using stairs was not possible or deemed unsafe beforehand. Patients 1 and 2 showed similar results, with shorter times required to ascend and descend stairs at T5 (42.5 s and 43.5 s) compared with T4 (49.7 s and 47.1 s), corresponding to improvements of approximately 7 s and 4 s, respectively. Patient 3 needs longer to overcome the stairs, with a time needed remaining constant at 72 seconds for T4 and T5 (**Figure 4**).

### Balance Confidence and Functional Independence

The ABC-D score is reported for T3, T4 and T5 and depicts fluctuations throughout all three patients over each documented visit (**Figure 5**). The Activities-Specific Balance Confidence Scale ranges from 0 to 100, where 100 equals full confidence. While the score constantly improves for patient 1 rising from 40 to 60, patient 2 and 3 depict an improvement from T3 to T4 followed by a decrease from T4 to T5. Patient 2 reports similar scores compared to patient 1, ranging from 45 to 60, however patient 3 reports lower scores between 9 and 30 (**Figure 5**).

Similarly, the LCI-5 (Locomotor Capabilities Index), depicted in %, revealed a marked increase in mobility levels across the study period. Patient 1 and 2 reached and LCI-5 of nearly 80% by the end of the observation, increasing by 40% compared to T3. Patient 3 reported a final LCI-5 of 40% at T5, 20% higher than the first observation at T3 (**Figure 5**).

### Prosthesis Usability

Another notable result was observed in the System Usability Scale (SUS), which also changed over the course of the timepoints, suggesting shifts in perceived usability of the prosthetic technology. This score also ranges from 0 to 100 with 100 representing the best imaginable usability. Patient 1 reported a slight decrease in usability from T3 (78) to T5 (60) and patient 3 a more notable decrease in usability (from 78 to 38), however patient 2 experienced an increase in usability from T3 (73) to T5 (80) (**Figure 6**).

The subjective score Q-TFA was also only documented from T3 to T5 and ranges from 0 to 100. It is separated into four sub-scores: use, mobility, problems and global health. The Prosthetic Use Score describes the time that the prosthetic is worn. A use score of 100 indicates that the prosthesis is worn daily for more than 15 hours. The Prosthetic Use Score depicts parallel patterns throughout each of the three patients. Where the reported score for T3 to T4 rises noticeably, the score remains constant from T4 to T5 (**Figure 7**).

The Q-TFA Mobility Score describes the prosthesis user’s ability to move, change and maintain postures when using the prosthesis with a score of 100 indicating the best possible mobility. This score remains relatively constant from T3 to T5 with slight improvements documented for patient 1 and 2 and a slight decrease in patient 3 (**Figure 7**).

The Problem Score, as part of the Q-TFA, captures the extent of specific problems related to the amputation and prosthesis itself, as well as the impact these problems have on the patient’s quality of life. Here a higher score indicates more serious problems. The score remains relatively constant throughout T3 to T5, with values ranging from 41 to 21. Patient 1 reports less problems from T4 to T5 (**Figure 7**).

The Global Health Score describes the patient’s overall perception of the current situation, with 100 indicating the best possible overall situation. This score slightly improves from T3 to T5 for patient 2 and 3, whereas remaining relatively constant for patient 1 as T3 is missing (**Figure 7**).

## DISCUSSION

This exploratory pilot study investigated the clinical outcomes associated with the early use of the Össur Power Knee as the primary prosthesis during rehabilitation after unilateral transfemoral amputation and found improvements in patient-reported pain, mobility, quality of life, and functional outcomes over a five-month follow-up period. To what extent these findings can be attributed to the powered knee will be discussed in the following paragraphs.

### Pain Assessment

A steady reduction in pain on the patient’s amputated side was observed, with NRS scores decreasing in patients 1 and 2. This suggests not only successful prosthetic fitting but also an encouraging level of user adaptation over time. Pain on the intact limb also improved in patient 1 and 2, although not to the same extent. The decline in pain levels points to the effectiveness of both device-based support and complementary rehabilitation efforts, including pharmacological pain management. Patient 3 reported a slight increase in pain by 2 points (from 1 to 3), however experienced pain also rose in the non-amputated leg from 0 to 2 throughout the observation period. Thus, the cause of this reported increase cannot be solely attributed to the Össur Power Knee and should be investigated additionally.

When compared to pain levels after three months reported by transfemoral amputees who received MPKs and NMPKs, the average score is similar between all three groups.^3^ While the MPK group reported an average pain intensity of 2.6 (n = 9) after three months, the NMPK group reported a mean pain intensity of 2.3 (n = 6), lastly the patients observed in this study, who received a powered knee reported a mean pain intensity of 2 (n = 3) after three months. The difference in reported pain between MPK and NMPK patients was not significant, whether the reported pain differs significantly between these two groups and patients who received a powered knee should be examined with a larger sample size.

It is also notable, that pain usually decreases after prosthetic fitting and early rehabilitation, regardless of the specific prosthesis used.^3,6^ Therefore, attributing the pain reduction solely to the Össur Power Knee cannot be justified within this study's circumstances.

### Health-related Quality of Life

Subjective health outcomes were similarly positive. EQ5D-5L scores increased amongst all three patients suggesting improvements in key domains such as pain management, mobility, and emotional well-being. These changes may reflect an enhanced sense of independence and improved reintegration into daily life, aspects that will be explored in greater detail in the following sections.

It is to note, that patient 3 reported consistently lower EQ5D scores from the baseline onwards, compared to patient 1 and 2, in line with higher perceived pain, lower mobility scores and possibly older age. This indicates that individual clinical characteristics and functional limitations may substantially influence subjective outcome measures.

In broader context, previous studies have shown that users of MPKs generally report higher health-related quality of life compared to users of NMPKs.^3,23^ In their systematic literature review Thibaut et al., discovered that all studies using the SF-36 reported better results for MPK users than NMPK users regarding their QoL.^23^ While the present findings for powered knee users are limited by the small sample size, the observed improvements across all participants suggest that powered knee systems may achieve at least comparable benefits to MPKs in terms of patient reported QoL.

Furthermore, when compared to reported EQ5D-5L scores of experienced transfemoral amputees, who report a median QoL of 77.8 with a prosthesis (n = 215),^24^ our patients reported a mean EQ5D-5L of 71, at T4, and 87.5 at T5 (patient 3 missing). Subsequently the health-related quality of life of powered knee users only five months after rehabilitation appears to be comparable to, or even exceeding, values reported in more experienced prosthesis users. This interpretation should be approached with caution due to the small sample size.

### Objective Mobility and Functional Capacity

Functional mobility showed objective measurable gains in all participants. An improvement is reflected by rising AMPPRO, 10MWT, TUG, 2MWT and TST scores. With patients 1 and 2 reaching values typically associated with high mobility and patient 3 presenting moderate mobility. Performance tests showed improvements in walking speed, stability, and endurance. Although these results were stagnant in patient 3, their AMPPRO score rose. The fact that powered knee users maintained or improved their performance in these tests without regression suggests that powered components may help compensate for lost muscular control in high-demand activities. These functional gains are particularly notable given that all patients began rehabilitation without prior experience using a prosthesis. That such progress was achieved within a relatively short timeframe underscores the potential benefit of introducing powered components early in the rehabilitation process.

In their study Morgan et al., examined functional outcomes of MPK and NMPK users after transfemoral amputation.^3^ After three months NMPK users reached an AMPPRO score of 27.5 (n = 6) and MPK users 35.9 (n = 9). In our study, power knee users reached an average AMPPRO score of 33 after three months, which clearly surpasses the NMPK users and is comparable to the mobility predictor of the MPK users. At the same time, the NMPK users performed the TUG test in 40.7 (n = 6) seconds and the MPK users in 15.9 seconds (n = 9). After three months our patients needed an average of 30.8 seconds to complete this test, performing better than the NMPK users but not as good as the MPK users, yet these results are to be compared with caution due to the small sample sizes.

It is also notable, that our three patients were able to ascend and descend stairs without aids three months after fitting with the Össur Power Knee. In addition, only two minor falls without injury and unrelated to the device occurred during follow-up procedures.

Whether a powered knee prosthesis outperforms MPKs in early rehabilitation cannot be confirmed nor denied. Further studies with larger sample sizes are necessary to determine significant differences. However, an increase in mobility within the first five months of rehabilitation in transfemoral amputees fitted with a powered knee was depicted with this study. Major improvements in basic mobility functions are portrayed by improvements in gait speed, agility and balance as seen in the tests mentioned above. Pasquina et al.,^6^ reported in a case series of four soldiers with combatrelated transfemoral amputation who received the Power Knee as their initial prosthesis, that the patients achieved mobility milestones considerably faster than historically reported for other prosthetic knee users. Gait biomechanics and functional performance were comparable to those of experienced microprocessor knee users, and preliminary evidence suggested a reduction in contralateral joint loading. These results support the notion that the Power Knee can facilitate early gains in mobility and functional independence.

### Subjective Mobility

Subjective mobility scoring systems include the LCI-5 and ABC-D. The LCI-5, which reflects confidence in performing locomotor tasks such as getting up from a chair, walking and using ramps and stairs also rose amongst all three patients from T3 to T5. By T5 patients reached scores up to 80.36% with an average LCI-5 of 66.67%. This alignment between performance and perception adds robustness to the data, indicating that patients not only performed better but also felt more capable. When compared to MPK and NMPK users, who reached and LCI-5 of 74.46% and 68.93%,^25^ our patients reported locomotor capabilities similar to NMPK users, with some patients reaching similar values to those reported by MPK users.

However, changes in ABC-D scores were more variable. Where patient 1 depicted a constant increase in balance from T3 to T5, patient 2 and 3 both reported the highest activity-specific balance confidence score at T4, which contradict with the improvements in objective mobility from T3 to T5. The ABC-D scores of patients 1 and 2 at T5 can be interpreted as a moderate level of physical functioning, whereas Patient 3 depicts a low level of physical functioning.^19^

### Prosthesis Usability

The SUS and Q-TFA depict the subjective prosthesis usability over time. While the SUS starts out relatively high at T3, reaching a mean of nearly 80 and remains at that level at T4, the score drops noticeably by T5, with patient 1 reporting a SUS of 60, patient 2 80 and patient 3 37.5, leading to a mean SUS score of 59 at T5. While the reported SUS of the Power Knee can be interpreted as nearly excellent at T3 and T4, it drops to a poor level of usability at T5, that can be interpreted as a system with significant usability issues.^21^ This decline in subjective usability may be related to the increasing functional demands placed on the Power Knee as rehabilitation progresses and patients transition to more complex and variable real-world tasks. In this phase, initial positive impressions may give way to a more critical evaluation of the system’s performance in everyday situations. In addition, rising user expectations, growing awareness of the prosthesis’ limitations and potential discrepancies between anticipated and actual performance may contribute to lower usability ratings. The variability between the users, as reflected by the wide spread SUS scores at T5, suggests that adaptation to the device and perceived usability are highly patient-specific.

This is inconsistent with the Q-TFA scores reported by the patients from T3 to T5. The reported prosthesis use over time rose notably from T3 (mean = 31) to T4 (mean = 63) and remained at that level at T5. At the same time, patients 2 and 3 reported the same problem score at T3 and T5, while patient 1 even reported the least problems at T5. In their systematic literature review of Q-TFA scores in transfemoral amputees using socket protheses with follow-up periods ranging from one to ten years, the authors Tan et al.,^26^ reported a mean global health score of 41.57 (n = 245). This value is slightly lower than the global health score observed in the present cohort of three Power Knee users, which averaged 44.45. Regarding prosthetic mobility according to the Q-TFA, the literature reports a mean score of 54.94 (n = 128),^26^ whereas the Power Knee users in this study demonstrated a considerably lower mean mobility score of 37.43, yet only after five months of use. This discrepancy may reflect the relatively short adaptation period in our cohort, suggesting that mobility-related outcomes may require longer exposure to powered prosthetic systems before reaching a plateau comparable to that observed in long-term conventional socket prosthesis users. In contrast, prosthetic use scores were higher in the present study. The reviewed literature reports an average prosthetic use score of 49.68 (n = 183),^26^ whereas the Power Knee users achieved a mean score of 62.37. This indicates a higher self-reported frequency or intensity of prosthesis use in the powered knee cohort despite lower mobility scores, which may suggest improved acceptance or increased reliance on the device in daily life. Finally, prosthesis problem scores were broadly comparable between groups. Conventional socket prosthesis users reported a mean score of 37.74 (n = 128)^26^ while Power Knee users reported a slightly lower mean score of 33.63, indicating marginally reduced perception of prosthesis-related problems in the powered knee cohort.

### Early Rehabilitation and Training Prostheses

In line with the findings of Pelzer et al.,^27^ who demonstrated that using a training prosthesis (TP) was associated with a significantly shorter rehabilitation stay, our data suggest that the early use of a powered knee can similarly contribute to efficient rehabilitation progress. While Pelzer focused on structural aspects of early prosthetic provision, our results add functional and user-reported improvements to the findings.

This perspective is reinforced by Morgan et al.,^3^ whose pilot randomized controlled trial showed improved mobility and balance confidence in early MPK users compared to those using non-microprocessor knees.^14^ Our findings align with theirs, suggesting that motorized prostheses, when integrated early in care, may possibly lead to faster functional recovery especially when paired with targeted training.

### Limitations

This exploratory case series has several limitations. The small sample size (n = 3) limits statistical power and generalizability. Furthermore, the absence of a control group prevents causal attribution of observed improvements to the powered knee. At the same time, patient heterogeneity introduces variability, while the five-month follow-up may not capture long-term outcomes. Incomplete data due to fatigue, medical constraints or incomplete questionnaires may have affected result reliability. Subjective outcome measures (e.g., ABC-D, SUS) may be influenced by individual expectations and psychological factors. Finally, the safety of the powered knee is only judged by AEs and SAEs. Further data, including detailed documentation during rehabilitation or even qualitative patient interviews would be necessary to further evaluate the safety and feasibility of a powered knee. These limitations highlight the need for larger, controlled trials to validate the preliminary findings.

## CONCLUSION

This explorative pilot study examined clinical outcomes associated with the early use of the Össur Power Knee during rehabilitation in individuals with transfemoral amputation. Within the first five months after prosthesis fitting, improvements were observed across functional mobility, prosthetic use and quality of life supported by both objective and patient-reported measures. These findings indicate that early rehabilitation with a powered prosthetic knee is reasonable and can lead to measurable clinical benefits.

At the same time, variability in patient-reported outcomes such as pain and system usability suggest that adaptation to powered knee technology is highly individual and may depend on factors such as prior mobility level, training intensity and user-specific needs. This underlines the importance of a tailored rehabilitation approach when introducing advanced prosthetic systems at an early stage of recovery.

While the results are encouraging, the small sample size and exploratory design limit the strength of the conclusion. Future research should build on these findings through larger controlled studies, with particular attention to optimizing training protocols and identifying which patient groups benefit most. Long-term follow-up will also be necessary to determine whether early gains translate into sustained functional and clinical advantages.

## Figures and Tables

**Table:** 

Assessment	Yes	No
**NRS with & without Prosthesis**		
**EQ5D-5L**		
**AMPPRO**		
**10m Walk Test**		
**2 min Walk Test**		
**Timed Up and Go Test**		
**Timed Stair Test**		
**ABC-D**		
**LCI-5**		
**SUS**		
**Q-TFA**		

## APPENDIX: Case Report Form: Power Knee Baseline

Patient-ID: _______________________ Date: _________________________ Study Visit No.: ______________________

Gender:

◯ Male
◯ Female
◯ Intersex

Date of birth: _________________

Height in cm: ___________________

Weight in kg: _____________________

Date of Amputation: __________________

Amputation Level: ____________________

Cause of Amputation:

◯ Trauma
◯ Vascular Disease
◯ Tumor
◯ Infection
◯ Other, please specify:
   _____________________________________________________________________________________________

Condition of the Contralateral Limb:

________________________________________________________________________________________________________________

__________________________________________________________________________________________

Medical History:

________________________________________________________________________________________________________________

__________________________________________________________________________________________

Date of Initial Prosthetic Fitting: _________________

Rehabilitation Goals:

________________________________________________________________________________________________________________

Patient Informed Consent Signed:

◯ Yes
◯ No—> Enrollment is not possible before consent has been obtained.

Assessments Performed:

Please indicate whether any adverse events have occurred since the initial prosthetic fitting.

Adverse Events (AEs):

◯ Skin irritation, pressure sores, or blisters at the socket–prosthesis interface
◯ Pain or swelling of the residual limb
◯ Unusual muscle soreness or fatigue during rehabilitation
◯ Minor falls or stumbling incidents without injury, regardless of whether they were related to the prosthesis
◯ Complaints or difficulties adapting to the prosthesis
◯ Technical or mechanical problems without serious consequences (e.g., instability or inadequate fit)

Serious Adverse Events (SAEs):

◯ Falls, regardless of whether they are related to the prosthesis, resulting in fractures, head injuries, or hospitalization
◯ Severe skin damage or infections requiring medical treatment or surgical intervention
◯ Prosthesis malfunctions resulting in significant injury
◯ Any other events, regardless of whether they are related to the prosthesis, resulting in hospitalization, permanent disability, or lifethreatening complications

If yes, please indicate whether the event was:

◯ Related to the prosthesis
◯ Unrelated to the prosthesis

Date of the Event: ____________________

Additional Comments on the Event:

________________________________________________________________________________________________________________

__________________________________________________________________________________________

_____________________________________________

Physician’s Signature
